# A diagnostic cascade from cholecystitis to Meckel’s diverticulum rupture: a case report

**DOI:** 10.1097/RC9.0000000000000951

**Published:** 2026-09-08

**Authors:** Lotfollah Abedini, Taha Negahdari, Sepehr Moradi, Reza Hosseinpour

**Affiliations:** a Department of General Surgery, Yasuj University of Medical Sciences, Shahid Jalil Hospital, Yasuj, Iran; b Department of Medicine, Shiraz University of Medical Sciences, Shiraz, Iran; c Breast Surgery Division, Department of Surgery, Yasuj University of Medical Sciences, Shahid Jalil Hospital, Yasuj, Iran

**Keywords:** acute abdomen, diagnostic laparoscopy, ectopic gastric mucosa, laparoscopic cholecystectomy, Meckel’s diverticulum, perforation

## Abstract

**Background:**

Meckel’s diverticulum (MD) is the most common congenital anomaly of the gastrointestinal tract, yet it rarely becomes symptomatic in adults. Perforation is an uncommon and often overlooked complication that may mimic more frequent abdominal conditions, particularly in the postoperative setting. MD presenting shortly after unrelated laparoscopic surgery is exceptionally rare and can pose a significant diagnostic challenge.

**Case presentation:**

A 36-year-old woman presented with acute right upper quadrant pain and was diagnosed with calculous cholecystitis. She underwent laparoscopic cholecystectomy and was discharged after an uncomplicated recovery. On postoperative day 3, she returned with abdominal distension, bilious vomiting, and persistent pain. Imaging revealed a port-site hematoma but no clear source of obstruction or leak. Due to sustained leukocytosis and clinical deterioration, diagnostic laparoscopy was performed, revealing bilious peritonitis and a necrotic, perforated MD with terminal ileal herniation. Diverticulectomy was completed, and the patient recovered fully.

**Discussion:**

Perforated MD can closely mimic postoperative ileus, small bowel obstruction, or biliary pathology, leading to delayed diagnosis. Imaging modalities frequently fail to detect this condition, underscoring the limitations of relying solely on radiologic evaluation when symptoms persist. Although a direct causal link to recent laparoscopic surgery cannot be confirmed, postoperative mechanical and perfusion-related changes may contribute to diverticular compromise in susceptible individuals.

**Conclusion:**

This case highlights the need to consider MD complications in patients who deteriorate unexpectedly after abdominal surgery. When clinical findings are discordant with imaging, early diagnostic laparoscopy remains essential for accurate diagnosis and timely intervention.

## Introduction

Meckel’s diverticulum (MD) is the most prevalent congenital anomaly of the gastrointestinal tract, arising from the incomplete regression of the embryonic vitelline duct^[^1,2^]^, with reported prevalence estimates ranging from 0.3% to 2.9% across study populations^[^3^]^. Meckel’s diverticula are usually clinically silent in adults, so they may be identified incidentally during a laparotomy performed for an unrelated reason^[^4^]^.

HIGHLIGHTS

- Meckel’s diverticulum may rarely perforate in the early postoperative period after unrelated laparoscopic procedures.
- Post-cholecystectomy abdominal pain with bilious vomiting can mimic ileus or bile leak, but may represent perforated Meckel’s diverticulum.
- Diagnostic imaging is frequently inconclusive; early diagnostic laparoscopy is critical in the setting of persistent postoperative deterioration.
- Pneumoperitoneum and postoperative bowel dynamics may precipitate perforation of vulnerable Meckel’s diverticula.
- This case emphasizes maintaining a broad differential diagnosis when evaluating unexpected postoperative abdominal sepsis.

The lifetime risk of complications from MD is reported to be approximately 4%, with bleeding, intestinal obstruction, diverticulitis, and, less commonly, perforation constituting the main clinical entities^[^5^]^. The pattern of presentation differs between children and adults: hemorrhage predominates in the pediatric population, whereas obstruction and inflammation are more typical in adults^[^4^]^. Systematic reviews from the 21st century confirm that, despite advances in imaging and minimally invasive surgery, the epidemiology and spectrum of presentation remain broadly unchanged, and MD continues to present as a diagnostic challenge^[^1^]^.

Perforation of MD is rare and may result from peptic ulceration due to ectopic gastric mucosa, foreign bodies, axial torsion, or spontaneous inflammatory necrosis^[^6^]^. Several case reports describe MD perforation mimicking acute appendicitis or generalized peritonitis, often diagnosed only at laparoscopy or laparotomy^[^7^]^. However, perforated MD presenting in the early postoperative period after unrelated laparoscopic procedures, such as laparoscopic cholecystectomy, is exceedingly uncommon. In this report, we describe a patient who underwent laparoscopic cholecystectomy for acute calculous cholecystitis and re-presented on postoperative day 3 with abdominal distension, bilious vomiting, and peritonitis, and was ultimately found to have a perforated MD^[^2^]^. This case highlights the need to consider MD-related pathology in the differential diagnosis of acute abdomen and sepsis following otherwise uneventful abdominal surgery^[^7^]^. This case report has been prepared and presented in accordance with the SCARE 2025 guidelines^[^8^]^.

## Case presentation

A 36-year-old woman with no significant past medical history presented to the emergency department on 15 September 2025 with a 4-hour history of right upper quadrant abdominal pain. Physical examination revealed a positive Murphy’s sign and bilateral upper quadrant tenderness, without guarding or rebound tenderness. She also reported several days of acholic stools. Initial laboratory and imaging evaluations identified a 12-mm gallstone and leukocytosis (15 000/µL). With a diagnosis of calculous cholecystitis, she underwent laparoscopic cholecystectomy. Postoperatively, she was monitored for 2 days, received appropriate prophylactic antibiotics, tolerated oral intake, and was subsequently discharged.

On 18 September 2025, she returned to the emergency department with recurrent right upper quadrant pain, nausea, bilious vomiting, abdominal distension, and an inability to tolerate oral intake, along with a history of obstipation. She was admitted with suspected ileus, and both nasogastric and rectal tubes were placed. Abdominal ultrasonography revealed a hematoma at the previous umbilical port site, while abdominal computed tomography (CT) without contrast enhancement or bowel-phase imaging was unremarkable; no radiologic evidence of Meckel’s diverticulitis was identified (Figs 1 and 2). Due to persistent severe abdominal pain, inability to ingest food, and marked leukocytosis (28 000/µL; the trend of serial white blood cell measurements throughout the patient’s clinical course is summarized in Table 1), exploratory laparoscopy was performed. Because of the reported port-site hematoma, the laparoscope was introduced through a subxiphoid port. Upon entry, a significant amount of bilious fluid was evacuated. The liver and extrahepatic biliary ducts appeared intact, and clip displacement was excluded.

**Figure 1.**
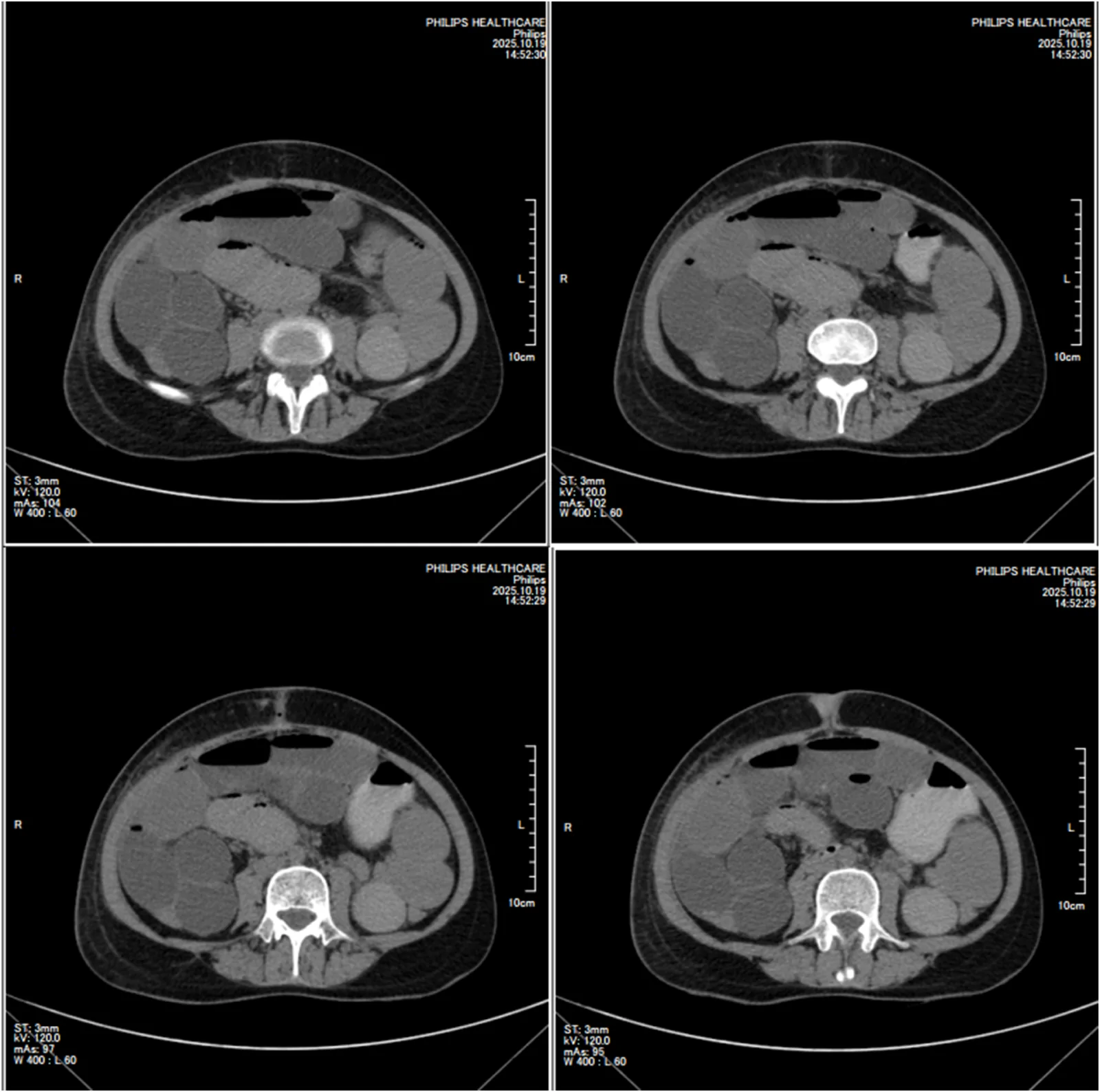
Axial abdominal CT image obtained during readmission, demonstrating mildly dilated bowel loops without radiologic evidence of inflammation or perforation of Meckel’s diverticulum, or small-bowel ischemia.

**Figure 2.**
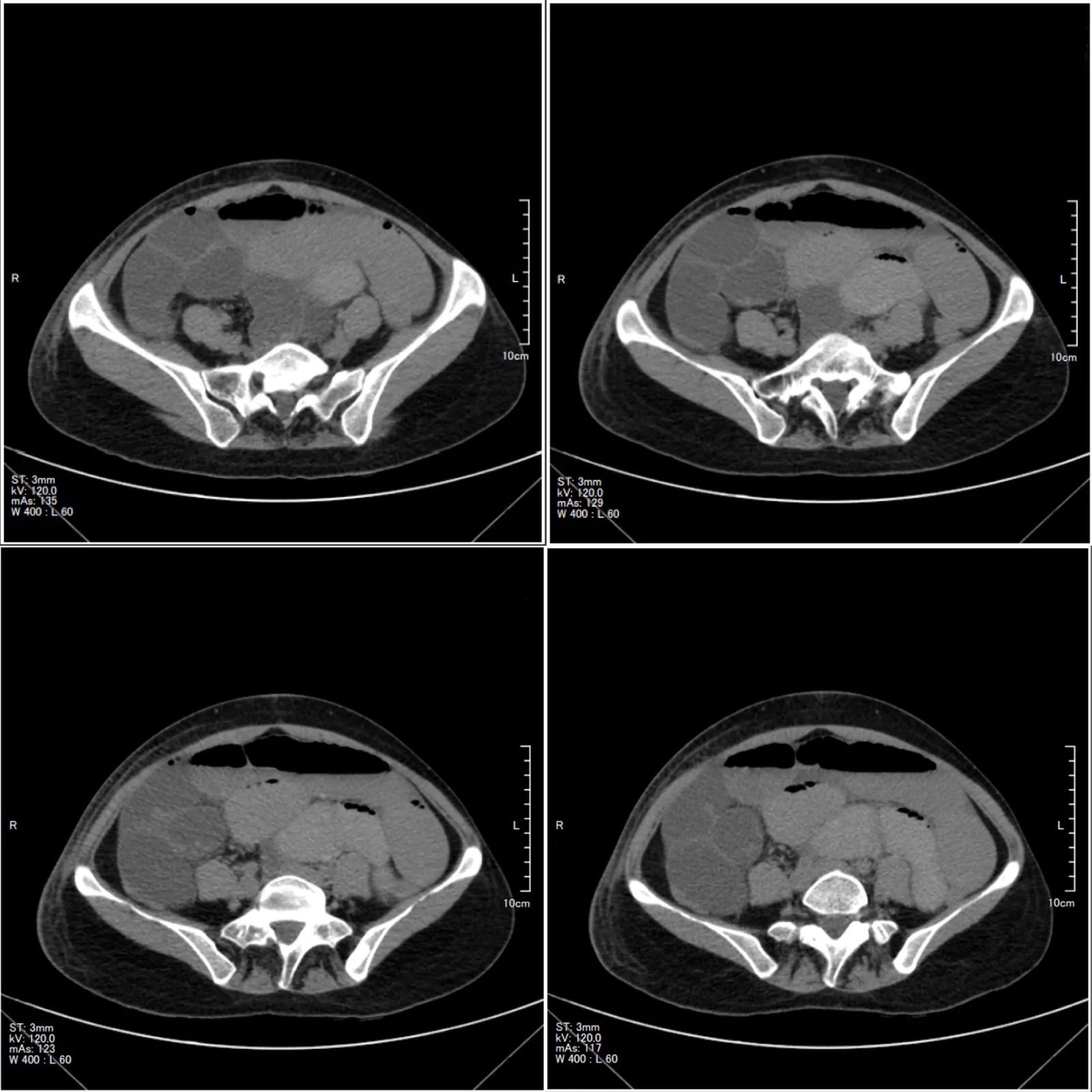
Axial pelvic CT image demonstrating the absence of a localized pelvic fluid collection or any identifiable inflammatory changes related to Meckel’s diverticulum.

**Table 1:** Serial white blood cell (WBC) measurements during the patient’s clinical course.

Clinical time point	Date (2025)	Clinical event	WBC (×10^3^/µL)
Initial admission (Preoperative)	15 Sep	Admission with acute calculous cholecystitis (before laparoscopic cholecystectomy)	15.0
Postoperative day 1	16 Sep	Early postoperative recovery after laparoscopic cholecystectomy	8.8
Readmission (Postoperative day 3, pre-re-exploration)	19 Sep	Readmission with abdominal pain, bilious vomiting, abdominal distension, and marked leukocytosis before exploratory laparoscopy	28.0
Post-diverticulectomy day 1	20 Sep	Early postoperative recovery	15.3
Post-diverticulectomy day 4	23 Sep	Clinical improvement	7.4
Post-diverticulectomy day 5	24 Sep	Continued recovery	8.3
Post-diverticulectomy day 6	25 Sep	Stable postoperative course	7.2

This table demonstrates initial leukocytosis at presentation, normalization after laparoscopic cholecystectomy, recurrent marked leukocytosis at readmission preceding exploratory laparoscopy, and progressive normalization following diverticulectomy.

Further exploration of the duodenum and ileum revealed an adhesion band in the terminal ileum. Closer inspection identified a necrotic, perforated Meckel’s diverticulitis located approximately 60 cm proximal to the ileocecal valve, with a neck base measuring 2 cm and a diverticular length of 4 cm. The terminal ileum was herniated into it in a loop-like fashion (Supplemental Digital Content Video 1, available at: https://links.lww.com/IJSCR/A144).

The presence of ectopic mucosa was suspected, given the necrotic and perforated appearance of the diverticulum; however, definitive confirmation depended on histopathologic examination, which was unavailable at the time. The resection margins appeared viable without gross residual necrotic tissue. Stapled diverticulectomy was performed, followed by appropriate antibiotic therapy. The patient showed satisfactory recovery, tolerated oral intake after 2 days of observation, and was discharged with stable vital signs and without further symptoms. She has remained symptom-free for 2 months to date and is satisfied with the surgical outcome.

## Discussion

MD is present in approximately 2% of the population, yet only a small proportion becomes symptomatic during adulthood. Among adults, obstruction and inflammation dominate the clinical spectrum, while perforation remains distinctly rare and frequently misdiagnosed pre-operatively. This rarity contributes to diagnostic delay, especially when MD mimics more common abdominal conditions^[^4^]^.

In our patient, the initial presentation was entirely compatible with acute calculous cholecystitis, and the first laparoscopic cholecystectomy was uneventful with no signs of bile leak or injury^[^9^]^. The patient’s return on postoperative day 3 with abdominal pain, distension, bilious vomiting, and constipation naturally raised suspicion for postoperative ileus, small-bowel obstruction, or biliary complications. However, these symptoms can also be produced by a perforated MD, highlighting the need to maintain a broad differential diagnosis when unexpected deterioration occurs after abdominal surgery^[^4^]^.

A key consideration in this case is whether the MD perforation may have been temporally associated with physiologic changes induced by laparoscopic surgery. Pneumoperitoneum during laparoscopy is known to increase intra-abdominal pressure and transiently reduce splanchnic and mesenteric perfusion, particularly at higher insufflation pressures. Experimental and clinical studies have demonstrated reversible alterations in intestinal blood flow and bowel motility during pneumoperitoneum, although clinically significant intestinal ischemia after routine laparoscopic cholecystectomy remains rare^[^10,11^]^. In the present case, no direct evidence of mesenteric ischemia or intraoperative bowel injury was identified. Therefore, any relationship between pneumoperitoneum and subsequent MD perforation remains hypothetical rather than causal. Nevertheless, in a diverticulum with pre-existing subclinical inflammation, a narrow neck, or compromised vascular supply, transient postoperative perfusion changes could theoretically contribute to localized ischemia or increased intraluminal pressure, potentially predisposing to delayed perforation. To our knowledge, direct evidence supporting this mechanism in MD is lacking, and the temporal association observed here should be interpreted cautiously^[^10,12^]^.

An alternative explanation is that the diverticulum may already have harbored subclinical diverticulitis or evolving ischemic injury before the cholecystectomy. Several published case reports have described spontaneous perforation of MD in the absence of direct traumatic or iatrogenic injury, suggesting that progressive inflammation, ectopic gastric mucosa-related ulceration, localized vascular compromise, or spontaneous transmural necrosis may independently result in delayed rupture^[^7,13^]^. In this context, the postoperative period may have merely coincided temporally with the natural progression of an already diseased diverticulum rather than representing the primary cause of perforation^[^4,14,15^]^. This possibility may also explain the initially unremarkable abdominal CT findings, as early or localized MD inflammation can remain radiologically occult prior to frank perforation^[^4,14^]^. Accordingly, although postoperative physiologic changes may theoretically have contributed to the clinical course, pre-existing occult diverticulitis with subsequent spontaneous necrosis remains a plausible alternative explanation and should be considered when interpreting the temporal association in this case.

Imaging studies were nondiagnostic – consistent with published literature showing that CT and ultrasound often fail to identify MD perforation pre-operatively. This reinforces the importance of early diagnostic laparoscopy in patients with persistent or worsening postoperative symptoms despite inconclusive imaging, as it remains the most reliable tool for both diagnosis and treatment. The second laparoscopy in our patient revealed intact biliary structures but identified a perforated MD with bile-stained peritonitis, confirming the true source of the clinical deterioration^[^9^]^.

Unlike foreign-body-related perforations or severe diverticulitis described in other case reports, our patient’s perforation appears more likely related to pressure-related or functional changes following surgery rather than an external insult. Clinically, the patient’s symptoms – distension, bilious vomiting, and obstipation – resembled postoperative ileus or small-bowel obstruction, yet the underlying mechanism was peritoneal irritation from MD perforation rather than mechanical obstruction^[^5^]^.

Overall, this case underscores several important principles: unexplained deterioration after a seemingly uncomplicated laparoscopic procedure warrants reconsideration of rare etiologies such as MD perforation; imaging may be misleading; and early re-exploration via laparoscopy can be decisive. It also reinforces existing literature suggesting that suspicious MDs encountered incidentally may merit resection to prevent catastrophic complications, especially those with a long or narrow base^[^1,4^]^.

## Conclusion

This case illustrates an uncommon but clinically significant presentation of perforated MD in the early postoperative period following an otherwise uncomplicated laparoscopic cholecystectomy. The overlap between postoperative ileus, biliary complications, and the manifestations of MD-related pathology can obscure timely diagnosis, particularly when imaging studies remain inconclusive. The patient’s rapid deterioration and the misleading preliminary evaluations highlight the importance of maintaining a broad differential diagnosis in patients who fail to recover as expected after abdominal surgery. Early diagnostic laparoscopy played a crucial role in identifying the true etiology and enabling definitive treatment. This report reinforces that although MD is often silent in adults, it should still be considered in the differential diagnosis of the acute abdomen and postoperative sepsis, and that prompt surgical exploration remains essential when clinical suspicion persists despite nondiagnostic imaging.

## Data Availability

All data relevant to this case report are included in the article. Additional information may be made available by the corresponding author upon reasonable request, subject to patient confidentiality and applicable ethical considerations.
